# Low-voltage driving high-resistance liquid crystal micro-lens with electrically tunable depth of field for the light field imaging system

**DOI:** 10.1038/s41598-022-21172-w

**Published:** 2022-10-19

**Authors:** Wenwen Wang, Wandi Chen, Yuyan Peng, Yongai Zhang, Qun Yan, Tailiang Guo, Xiongtu Zhou, Chaoxing Wu

**Affiliations:** 1 College of Physics and Information Engineering, Fuzhou University, Fuzhou, 350116 Fujian People’s Republic of China; 2 Fujian Science and Technology Innovation Laboratory for Optoelectronic Information of China, Fuzhou, 350116 Fujian People’s Republic of China

**Keywords:** Liquid crystals, Imaging and sensing, Applied optics

## Abstract

Light field imaging (LFI) based on Liquid crystal microlens array (LC MLAs) are emerging as a significant area for 3D imaging technology in the field of upcoming Internet of things and artificial intelligence era. However, in scenes of LFI through conventional MLAs, such as biological imaging and medicine imaging, the quality of imaging reconstruction will be severely reduced due to the limited depth of field. Here, we are proposed a low-voltage driving LC MLAs with electrically tunable depth of field (*DOF*) for the LFI system. An aluminum-doped zinc oxide (AZO) film was deposited on the top of the hole-patterned driven-electrode arrays and used as a high resistance (Hi-R) layer, a uniform gradient electric field was obtained across the sandwiched LC cell. Experimental results confirm that the proposed LC MLAs possess high-quality interference rings and tunable focal length at a lower working voltage. In addition, the focal lengths are tunable from 3.93 to 2.62 mm and the *DOF* are adjustable from 15.60 to 1.23 mm. The experiments demonstrated that the LFI system based on the proposed structure can clearly capture 3D information of the insets with enlarged depths by changing the working voltage and driving frequency, which indicates that the tunable *DOF* LC MLAs have a potential application prospects for the biological and medical imaging.

## Introduction

Light field imaging (LFI), as the most crucial and straightforward instrument, has been widely used as the medical diagnosis^1^, clinic examinations^2^, biological research^3^, material science^4^, education^5^ and other areas^6^ due to allow the investigations of cellular structures and their dynamics^7^. Compared with the traditional imaging method as the dominant approach to observe the matter is limited to recording only the two-dimensional (2D) aspect, whereas three-dimensional (3D) LFI can obtain information on the location and direction of spatial objects at the same time. With the rapid proliferation of high-speed imaging, continuous-depth cues, refocusing, volumetric display and head-mount holographic reconstruction, extensive efforts have been made to improve the quality of this modern imaging systems^8–10^. The LFI system can be constructed by implementing microlens arrays (MLAs) between the primary lens and the single camera, which captures the information of an object from different perspectives that emanate or reflect from a scenario^11–13^. The imaging manifestation is closely related to which the fundamental coupling between the size of the lens aperture and the depth of field (*DOF*), and the entire volume acquisition be deemed as a applicability method to integrates with various medicinal or biological imaging system across from face recognition to single-cell studies^14,15^.

Extensive study of *DOF* extension through imaging system based on the key MLAs has been performed^16,17^. Among them, the immediate approach to expand the *DOF* is scan the prototype by changing the focal length of the MLAs axially^18^. However, it can lead to uneven amplification and resolution problems in the LFI system. Unlike the traditional mechanical focusing method, Javidi et al. have proposed a way to significantly increase the *DOF* of the integral imaging propotype by reducing the numerical aperture of MLAs^19^. Orth et al. put forward a way to extended the *DOF* of images and reconstructed complex 3D objects with high spatial resolution by filtering out multicore optical fibers^20^. Nath et al. reported a multi-modal microscopic imaging system with high resolution on a single platform by using smartphone^21^. Yang et al. raised a aperture scanning fourier ptychographic microscopy that are emphasis on the 3D sample refocusing and experimental studies demonstrated that it has ability to refocus through thick samples at designated depth^22^. Although the high-quality 3D object information from angular and spatial dimensional can be clearly captured by changing the focal plane of the high-performance MLAs, whereas the rendered images has limited *DOF* frequently.

As to the modern LFI system, tunable and enlarged *DOF* is the most significant factor to determine the imaging properties. Therefore, a few researchers have employed the polymer dispersed liquid crystal (LC), axisymmetric prearrangement LC (APLC) and blue phase LC to fabricate the electrical-field-driven LC MLAs^23–25^. Specifically, among these three main LC types, the LFI based on APLC has a promising development prospect due to light field acquisition in a single photographic exposure, low cost, the focal plane of the imaging system can be change by regulating the driving voltage of LC MLAs applied to the LFI^26^. Compared with conventional MLAs by adjusting focal plane through mechanically moving the relative position of the lenses, electrically controllable LC MLAs can extend the *DOF* without any mechanical movement through electrically tunable focusing capability since polarization is an inherent property of LC materials, stray light vibrates along the optical axis of the LC MLAs with an electric vector^27–30^. Therefore, the LC MLAs has become optional to traditional MLAs in the modern LFI system due to a simple structure, light weights, low costs and tunable focal length^31–34^. Schechner raised a approach to extend the *DOF* by using utilize so-called polarization independent LC MLA with electrically tunable focal length^35^. Kwon et al. proposed a switchable bifocal polymer LC MLAs with polarization switching layer to further improve the *DOF* of the microscopy system^36^. Huang et al. reported a fast scanning multifocal LC MLAs to further extend the *DOF* instead of the fixed MLAs in the LFI system^37^. However, the *DOF* of the system expands from 62.5 to 436. 8 µm. Consequently, electrically LC MLAs has attracted a lot of attention due to its adjustable focal lengths for the LFI system^38–40^.

In this paper, we demonstrated that with a delicate design of the high-resistance LC MLAs (Hi-R LC MLAs), it is possible for capturing light field information of objects and improving the working range of the LFI system significantly. This optical structure is used to perform imaging operation by manipulates an incident beam of light by the main lens with adjusting working voltages and driving frequencies applied to the Hi-R LC MLAs. Compared with previous LC strcucture, the proposed AZO LC structure have tunable focal lengths with low-voltage driving and high-quality optical properties can be achieved due to the existence of Hi-R layer. Moreover, we further demonstrates the optical mechanism of low-voltage driving Hi-R LC MLAs, which significantly increased the viewing distance for LFI system, providing a promising approach for the potential applications of this study include medicine imaging and biology imaging.

## Results

### Optical images of the hole-patterned electrode arrays

**Figure 1:**
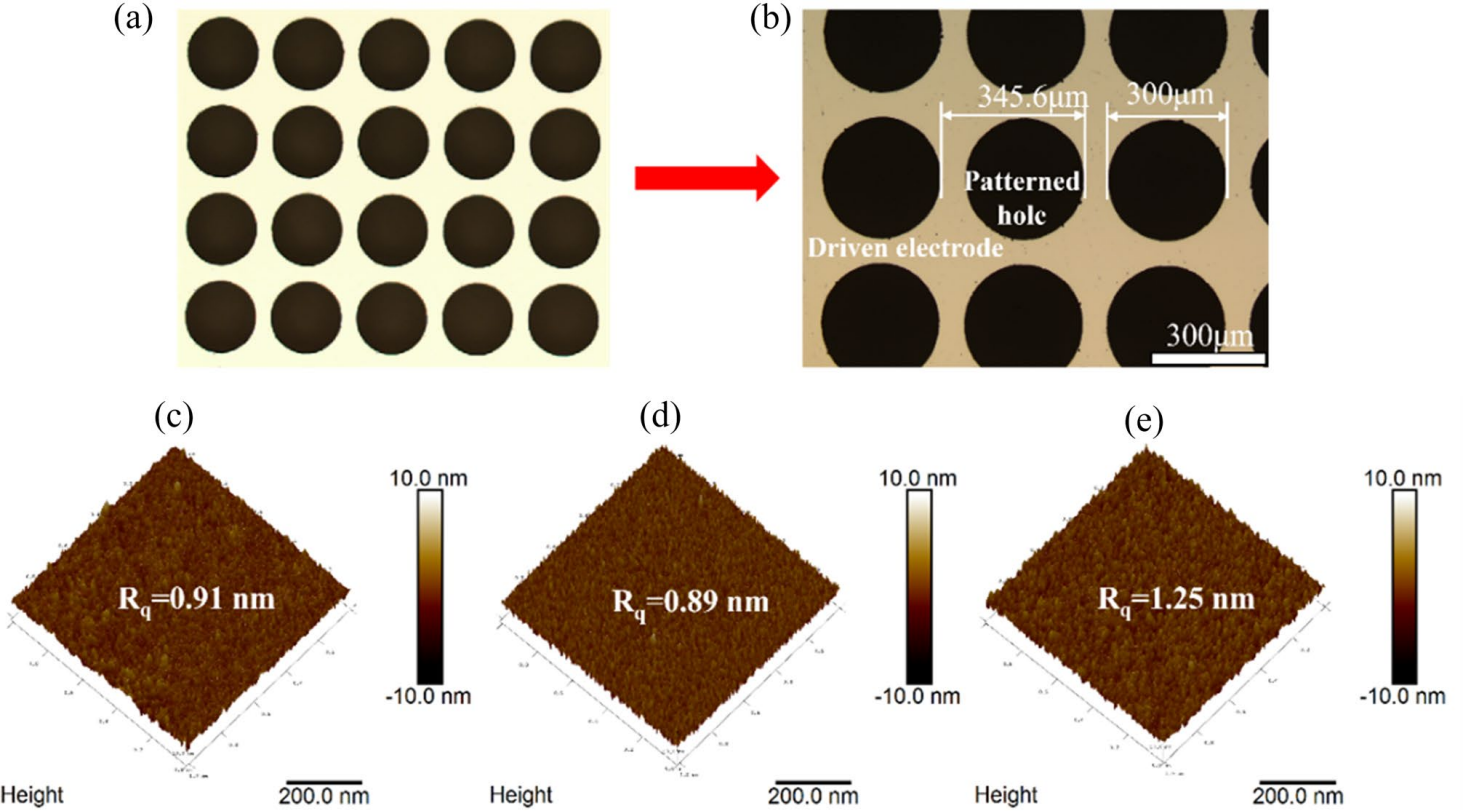
(**a**) Microscope image of the fabricated Hi-R electrode arrays. (**b**) Magnified microscope image of (**a**); and (**c**–**e**) 3D AFM images of AZO Hi-R layer, and their thickness are approximately 20 nm, 30 nm and 50 nm, respectively.

An optical microscope (Olympus, OLS 3000) was employed to observe the optical image of a hole-patterned driven-electrode arrays. The Al layer with hole-patterned arrays, which is uniformly coated on the top glass substrate as the electrodes, as schematically described in Fig. 1a. Furthermore, Fig. 1b presents that the diameter and the adjacent distance of a hole-patterned driven-electrode were 300 μm and 345.6 μm, respectively. The AZO Hi-R layer is deposited on the surface of aluminum film layer with hole-patterned arrays and the images of three-dimensional (3D) atomic force microscope (AFM) is revealed in Fig. 1c–e. It is apparent that the root-mean-square (RMS) roughness of Hi-R layer gradually become larger with the increases of the film thickness. Figure 1c shows that the RMS roughness of AZO Hi-R layer with ~ 20 nm thickness is 0.91 nm and the square resistance measured by the high resistance instrument (MCP-HT800) is much higher than ~ 10^11^ Ω/sq, indicating that the fabricated AZO Hi-R layer has a poor uniformity and electrical conductivity. For ~ 50 nm AZO Hi-R layer, the RMS roughness in Fig. 1e is 1.12 nm and the square resistance is approximately ~ 10^7^ Ω/sq, which indicate that the deposited AZO film layer has a good uniformity and conductivity by contrast to ~ 20 nm AZO Hi-R layer. However, as shown in Fig. 1d, the RMS roughness of ~ 30 nm AZO Hi-R layer is 0.89 nm and the square resistance is approximately ~ 10^8^ Ω/sq, indicating that the as-prepared AZO film has good uniformity, which is beneficial to form a gradient electric field distribution and obtain LC lens.

### Optical characteristics of Hi-R LC MLAs

**Figure 2:**
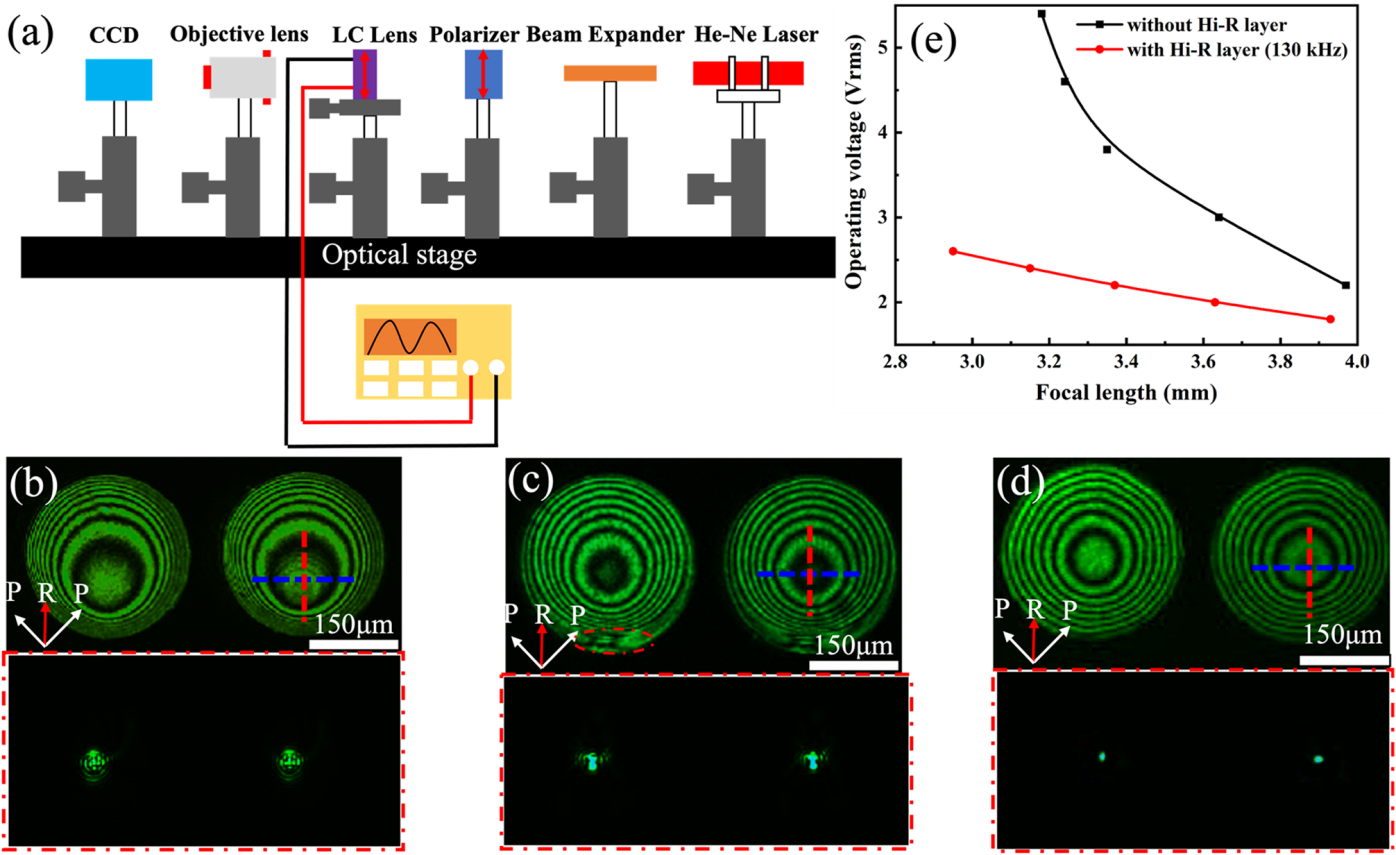
(**a**) The testing system for measuring the electro-optical properties of the LC MLAs; Optical properties of LC MLAs without Hi-R layer at (**b**) 2.2 *V*_rms_ and (**c**) 3.8 *V*_rms_; (**d**) Optical properties of Hi-R LC MLAs at 2.2 *V*_rms_ with driving frequency of 130 kHz; and (**e**) Focal length-dependent the working voltage for the different LC MLAs.

To further investigate electro-optical properties of as-prepared Hi-R LC MLAs, an automated self-assembled optical experimental setup with a position readout was built, as schematically in Fig. 2a. The collimated laser beam with wavelength of 632.8 nm from He–Ne laser device passes through the Hi-R LC MLA cell placed between two crossed polarizers, in which the rubbing direction of the Hi-R LC MLAs is oriented at 45° with respect to the transmission axis of these pair of crossed polarizers. Then, the laser beam was expanded and was normally incident through the polarizer and the Hi-R LC MLAs. Subsequently, the interference patterns occurs and the focal spot are measured with a magnified image captured by a CCD camera (6.45 × 6.45 µm), which is located behind the analyzer.

Figure 2b shows the measured interference patterns distribute nonuniformly and the focusing beam spots deviate from the center of LC MLAs with a voltage signal of 2.2 *V*_rms_, in which without AZO Hi-R layer. This phenomenon of eccentric circle might be related to the boundary effect of electric field. The LC MLAs will be influenced by the boundary effect because the LC molecules have the pretilt angle whose direction is determined by the polyimide (PI) rubbing when the driving voltage is not large enough. When the working voltage is increased to 3.8 *V*_rms_, the electric field can permeate into the center of the LC MLAs. So, the focus beam spots are become smaller and closer to the center of LC MLAs, as shown in Fig. 2c. However, because of the LC molecular reoriented in opposite directions under the role of fringe electric fields, the disclination lines occur the edge of the LC MLAs^41^. When a low working voltage of 2.2 *V*_rms_ with a driving frequency of 130 kHz are applied, the same number of interference patterns can be obtained compared with the LC MLAs structure without Hi-R layer inwhich the AZO Hi-R layer is 30 nm and the square resistance is 4.88 × 10^8^ Ω/sq, as described in Fig. 2d. Notably, the edges of interference patterns are orderly and the radius of focusing beam spots become smallest. This is because the square resistance can be evenly consumed by the working voltage, and the gradient electric field can permeate into the center region of LC MLAs effectively, assisted by the AZO Hi-R layer^37,42^.

To evaluate the relationship between the focal length versus the working voltage of LC MLAs, the analyzer is removed and the rubbing direction is placed behind the polarizer, which parallel to its transmission axis. Figure 2e illustrates the functional relationship between focal length and driving voltage of LC MLAs with or without Hi-R layer. It was noted that, the working voltage applied on the LC structure without Hi-R layer is much larger than those with Hi-R layer, especially when the focal length is 3.2 mm, the LC structure without Hi-R layer needs to apply 4.6 *V*_rms_, while the LC MLAs with Hi-R layer almost less than half of voltage that only needs to apply 2.4 *V*_rms_. And the range of the adjustable focal length of the Hi-R LC MLA is 0.99 mm, which is a bit higher than the adjustable focal length of the LC MLA of 0.79 mm. This mainly attributed to that almost all the LC molecules from the LC lens border to the lens centre are conducive to the focusing behaviour for the extraordinary ray and the refractive index distribution between the LC lens centre and the edge will become very small compared with traditional LC MLAs without Hi-R layer, and this means that the focal length of the lens changes to saturation with the increases of driving voltage by the assistance of Hi-R layer in the LC MLAs^42^.

For the Hi-R LC MLAs, the focal length value can be estimated based on the measured interference patterns, as shown below:

1 $$f = \frac{{{\text{R}}^{2} }}{{2{\text{N}}\uplambda }}$$

where *R* denotes radius of the circular electrode aperture on the top substrate, *N* denotes the number of the interference patterns.

**Figure 3:**
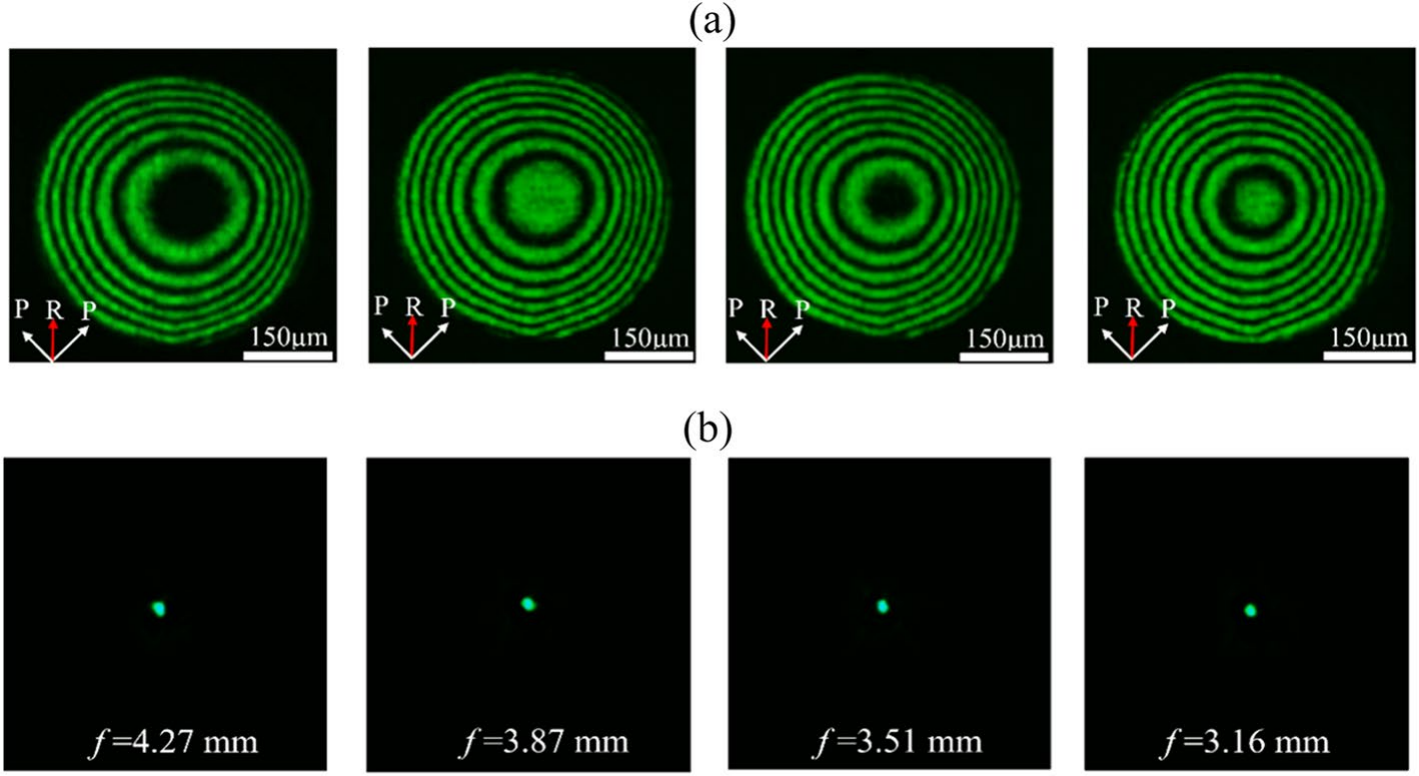
(**a**) The optical system for measuring interference patterns with applied frequency signals of 130 kHz at different working voltages of 1.8, 2.0, 2.2 and 2.4 *V*_rms_, respectively and the corresponding focusing beam spots (**b**).

Figure 3a illustrates the dependence of the interference patterns on the working voltage and Fig. 3b present the corresponding optical images of focusing beam spots with a frequency of 130 kHz. When the working voltage was 1.8 *V*_rms_, the fringes appeared gradually, but did not appear near the hole center of the hole-patterned driven-electrodes. This is because that the working voltage of LC deflection is still below the threshold voltage near the hollow hole, further causing LC molecules to not reorient in these regions. However, fine interference patterns are obtained near the hole center, which is due to the electric field is large enough to penetrate into the hollow hole when the working voltages is higher than 1.8 *V*_rms_. When the driving voltages is 1.8, 2.0, 2.2, and 2.4 *V*_rms_, Fig. 3a indicates that the numbers of interference patterns are 6, 6.5, 7 and 7.5 pairs, respectively. Furthermore, the spot sizes of focusing beam spots and focal length become smaller by the increases of the working voltages, as schematically depicted in Fig. 3b, and the theoretical spot size (PSF) are 10.99, 9.96, 9.03 and 8.13 mm according to the formula $\frac{{0.61\uplambda }}{{{\text{NA}}}}$. These results show that under the role of Hi-R layer, electric field can be efficaciously distributed in the hollow hole, which is conducive to the formation of high quality of Hi-R LC MLAs. Whereas it showed an off-centered lens profile, it mainly because that the electric field distribution of the LC lens array in the Hi-R layer isn’t separate into N parts evenly, and the resistance of each part isn’t equal in a single LC lens during the fabrication, which is deposited on the surface of each hole-patterned electrode. Furtherly, each node of the R–C circuit will produce an uneven gradient voltage distribution as a working voltage is supplied to the Hi-R LC MLAs. Therefore, the potential difference in the LC cell is easily generated an uneven parabolic electric field distribution which causes an off-centered lens profile.

**Figure 4:**
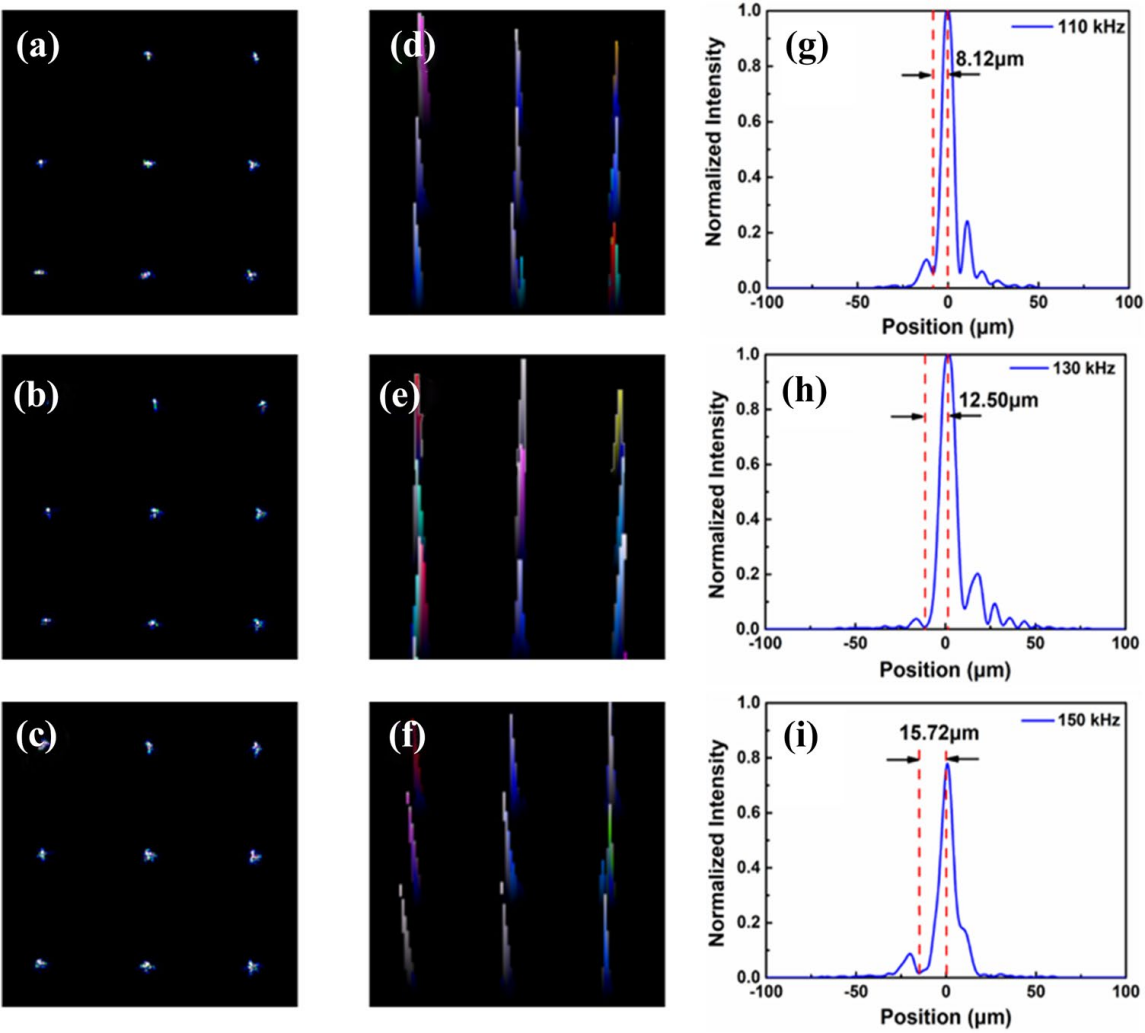
(**a**–**c**) 2D light intensity distribution for the driving frequencies of 110, 130, and 150 kHz at the standard working voltage of 2.2 *V*_rms_, respectively. (**d**–**f**) Corresponding 3D light intensity distribution; and (**g**–**i**) Normalised intensity distribution of a randomly selected focused spots.

To furtherly characterize the focal properties of driving frequencies defection, the focusing performances can be investigated with different driving frequencies 110 kHz, 130 kHz, 150 kHz of 2.2 *V*_rms_. The 2D light intensity distributions in Fig. 4a–c confirm that the light spots become smaller and the focus spots become sharper by increasing the frequency, and the relevant 3D light intensity distribution satisfies Gaussian distribution, as shown in Fig. 4d–f, and Fig. 4g–i shows the magnification image of a randomly selected focused spot. Thus, the effective diameters of focal spots are 8.1, 12.5, and 15.7 μm under the frequencies of 110, 130, and 150 kHz**,** accounting for 4.69%, 7.23% and 9.09% in regard to that of Hi-R LC MLAs, respectively. However, it can be seen that system has some non-symmetry ripple in PSF as illustrated in Fig. 4g–i, the asymmetric shapes might be attributed to the stray light caused by the reflected light from the substrates or the scattered light during the measuring. At the same time, the numerical aperture (NA) of the Hi-R LC MLAs changes from 0.035 to 0.047, and the low NA also induces the unsymmetrical side lobes in PSFs especially at the lower driving frequency case as shown in Fig. 4g–h. Based on the above measured results, indicating that the Hi-R LC MLAs has good converging ability and focusing characteristics under the control of driving voltage and frequency.

### Imaging performances of Hi-R LC MLAs

**Figure 5:**
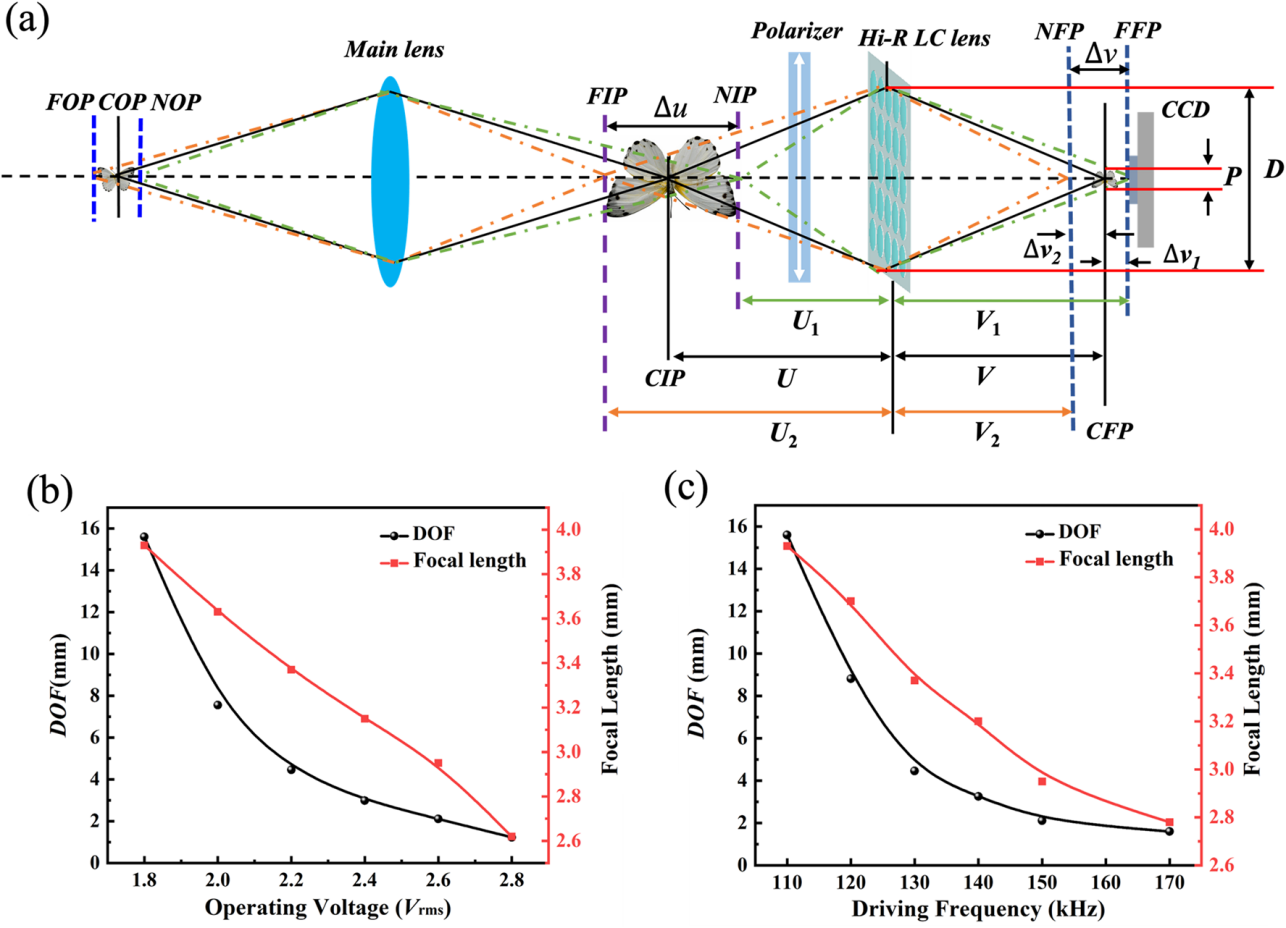
(**a**) The schematic illustration of the LFI system based on Hi-R LC MLAs; (**b**) the evolution of *DOF* and focal length with different operating voltage under the driving frequency of 130 kHz; and (**c**) the evolution of *DOF* and focal length with different driving frequency under the working voltage of 2.2 *V*_rms_.

The imaging schematic of the LFI system based on Hi-R LC MLAs depicted in Fig. 5a. When the Hi-R LC MLAs remains unchanged at a distance from the CCD camera, the depth of field (*DOF*) shifts along the optical axis synchronously. Therefore, the overall *DOF* will be significantly improved by changing the driving voltage of the Hi-R LC MLAs in the LFI system. The specimen is located in the central object plane (*COP*) and the light from the specimen can be converged through the main lens with the focal length of 50 mm at the center image plane (*CIP*), as schematically described in Fig. 5a. When the diffuse spot (*P*) is smaller than that the pixel of CCD camera, the object captured by CCD camera is used as a clear image. The certain plane is defined as the far focal plane (*FFP*) or the near focal plane (*NFP*), and the corresponding conjugate planes is used as the near image plane (*NIP*) or the far image plane (*FIP*). The focal plane of CCD camera is located in the center focal plane (*CFP*) and can obtain clear images at a conjugate plane when the specimen between the far object plane (*FOP*) and near object plane (*NOP*) passes through the Hi-R LC MLAs, and the distance $\Delta u^{\prime }$ between the *FOP* and *NOP* is defined as the *DOF* at object plane.

According to similar triangle relations, image distance *V*_*1*_ and *V*_*2*_ can be evaluated according to $V_{1}= V + \Delta V_{1} = \frac{DV}{{D - P}}$ and $V_{2}= V - \Delta V_{2} = \frac{DV}{{D + P}}$, respectively. Therefore, the corresponding object distances *U*_1_($U_{1}= \frac{fDV}{{VD - fD + fP}}$) and *U*_2_($\ U_{2} = \frac{fDV}{{VD - fD - fP}}$) are calculated according to the imaging formula of LC lens $\frac{1}{U} + \frac{1}{V} = \frac{1}{f}$ (*V* ≠ *f*), respectively. So, the distance *∆u* between *FIP* and *NIP* is defined as the *DOF* at image plane and the total *DOF* of Hi-R LC MLAs in the LFI system can be shown bleow:

2 $$\Delta u = U_{2} - U_{1} = \frac{{2f^{2} VDP}}{{(V - f)^{2} D^{2} - f^{2} P^{2} }}$$

where *V*_*1*_ is the image distance between lens and *FFP*, *V*_*2*_ is the image distance between lens and *NFP*, respectively. *V* is the image distance between lens and *CFP. ∆V*_*1*_ is the deviation distance between the *CFP* and *FFP* and *∆V*_*2*_ is the deviation distance between the *CFP* and *NFP*, respectively. *f* is the focal length of lens, *D* is the diameter and *P* is the aperture of the dispersion circle, respectively.

Figure 5b presents the driving voltage versus *DOF* of the Hi-R LC MLAs with the different focal lengths at a standard driving frequency of 130 kHz in the LFI system. The focal lengths changes from 3.93 to 2.62 mm and the corresponding *DOF* are shifted from 15.60 to 1.23 mm in the LFI system as the working voltages transferred from 1.80 *V*_rms_ to 2.80 *V*_rms_. Figure 5c depicts the functional relationship between driving frequency and *DOF* of the Hi-R LC MLAs with the different focal lengths under the voltage of 2.20 *V*_rms_. The focal lengths can be adjusted from 3.93 to 2.78 mm and the corresponding *DOF* are shifted from 15.60 to 1.60 mm as the driving frequencies varied from 90 to 170 kHz. Hence, the LFI system mentioned in our article is mainly used to verify that the proposed Hi-R LC MLAs can be used to observe the enlarged *DOF* of specimen by adjusting driving voltage and frequency. So, the *DOF* of specimen shall be the same as the LC MLAs which used for LFI system and the system *DOF* is at millimeter range. These results indicate that the prepared structure can be regulated by driving voltage and frequency effectively and has good imaging performance.

**Figure 6:**
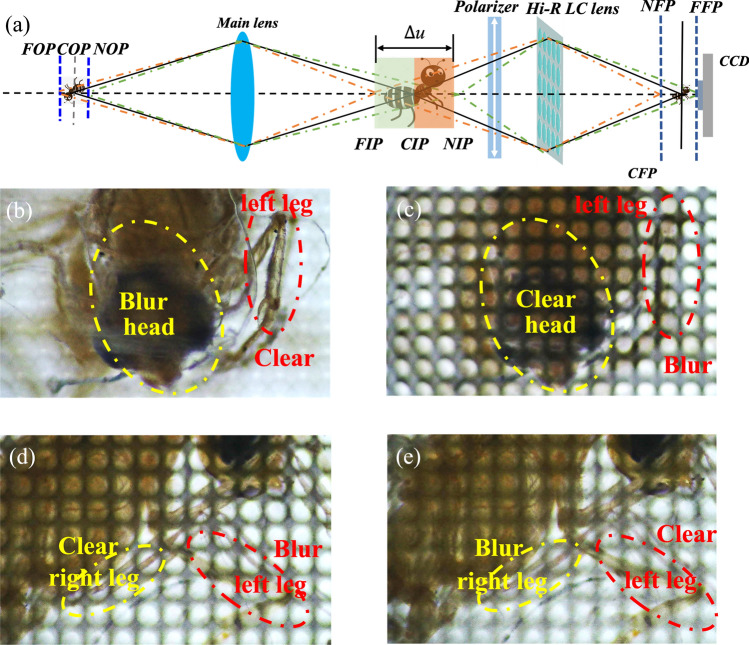
(**a**) Principle diagram of the LFI system based on Hi-R LC MLAs; Rendering images of the back leg of the insects in the LFI system with (**b**) *f* = 3.93 mm (1.9 *V*_rms_,130 kHz) and (**c**) *f *= 3.37 mm (2.2 *V*_rms_, 130 kHz); Rendering images of the front leg of insects in the LFI system with (**d**) *f* = 3.93 mm (2.2 *V*_rms_, 130 kHz) and (e) *f* = 3.70 mm (2.2 *V*_rms_, 120 kHz).

The principal diagram of the LFI system based on Hi-R LC MLAs is illustrated in Fig. 6a. The proposed LFI system consists of a CCD camera, a fabricated Hi-R LC MLAs, and a main lens with a focal length of 50 mm. In order to focus at the different depth regions, the focal length of Hi-R LC MLAs has been changed from 3.37 to 3.93 mm for respectively. The resolution of the CCD is 3840 × 2160 and the pixel pitch is 1.85 µm. The distance between the Hi-R LC MLAs and CCD camera is set as 5 mm. The light emitted from the ants is firstly concentrated in front of the main lens. And then, the image of the ant can be captured by the Hi-R LC MLAs and CCD cameras, where each micro-lens is considered as a small camera and can record the corresponding parts of the ant.

Figure 6b shows that the image of the left leg of the ant is near the focal plane of lens, and the ant's head is far from the focal plane, and the rendering image refocusing at the left leg (*DOF* = 15.60 mm) become clear in the LFI system as the focal length of Hi-R LC MLAs is 3.93 mm, in which the driving frequency of 130 kHz and working voltage of 1.9 *V*_rms_ are applied to the Hi-R LC MLAs. However, as show in Fig. 6c, the ant's head (*DOF* = 4.46 mm) become clear, and the left leg becomes blur on the rendering image from the LFI system of the Hi-R LC MLAs with the focal length of 3.37 mm as the driving frequency is fixed to be 130 kHz and the working voltage is transferred from 1.9 *V*_rms_ to 2.2 *V*_rms_. As Fig. 6d illustrated, the ant's right leg is near the focal plane of Hi-R LC MLAs, and the left leg of the ant is far from the focal plane, the light field image of Hi-R LC MLAs with the focal length of 3.37 mm (130 kHz, 2.2 *V*_rms_) is capable to render the right leg (*DOF* = 4.46 mm) as well image quality. Meanwhile, the same right leg is blur from light field image (*DOF* = 11.08 mm) of Hi-R LC MLAs with focal length of 3.70 mm (120 kHz, 2.2 *V*_rms_), as plotted in Fig. 6e. However, we can see that the resolution of the right leg in Fig. 6e is much lower than that of the right leg in Fig. 6d. By engaging rendering image from the following light field images, *DOF* of LFI system can be extended from 15.60 to 4.46 mm in insect space in front of the main lens by adjusting the working voltage and driving frequency applied to the Hi-R LC MLA.

## Conclusions

In summary, electrically Hi-R LC MLAs with low-working driving and high-quality interference rings have been successfully prepared, where AZO Hi-R layer are deposited on the hole-patterned electrode arrays. And then, Hi-R LC MLAs with tunable focal lengths and DOF are proved in the LFI system. The focal lengths of Hi-R LC MLAs are tunable from 3.93 to 2.62 mm and the corresponding DOF in the LFI system are adjustable from 15.60 to 1.23 mm with a low working voltage of 1.8*–*2.8 *V*_rms_ and driving frequency of 90–130 kHz. These results indicate that the optical performances of the proposed structure can be controlled effectively by shifting the voltages and frequencies. Moreover, Hi-R LC MLAs can precisely capture small-size specimens at different depth by effectively controlling working voltages and driving frequencies in the LFI system, and enlarged DOF of light field image scan be seen in elemental images. Based on these results, the low-voltage driving Hi-R LC MLAs with electrically tunable DOF can be expected to a promising tool for a potential application prospects in the fields of biology and medicine for the LFI system, the final processed long DOF light field image will further investigate in the future work.

## Experimental details

### Operating principles of Hi-R LC MLAs

**Figure 7:**
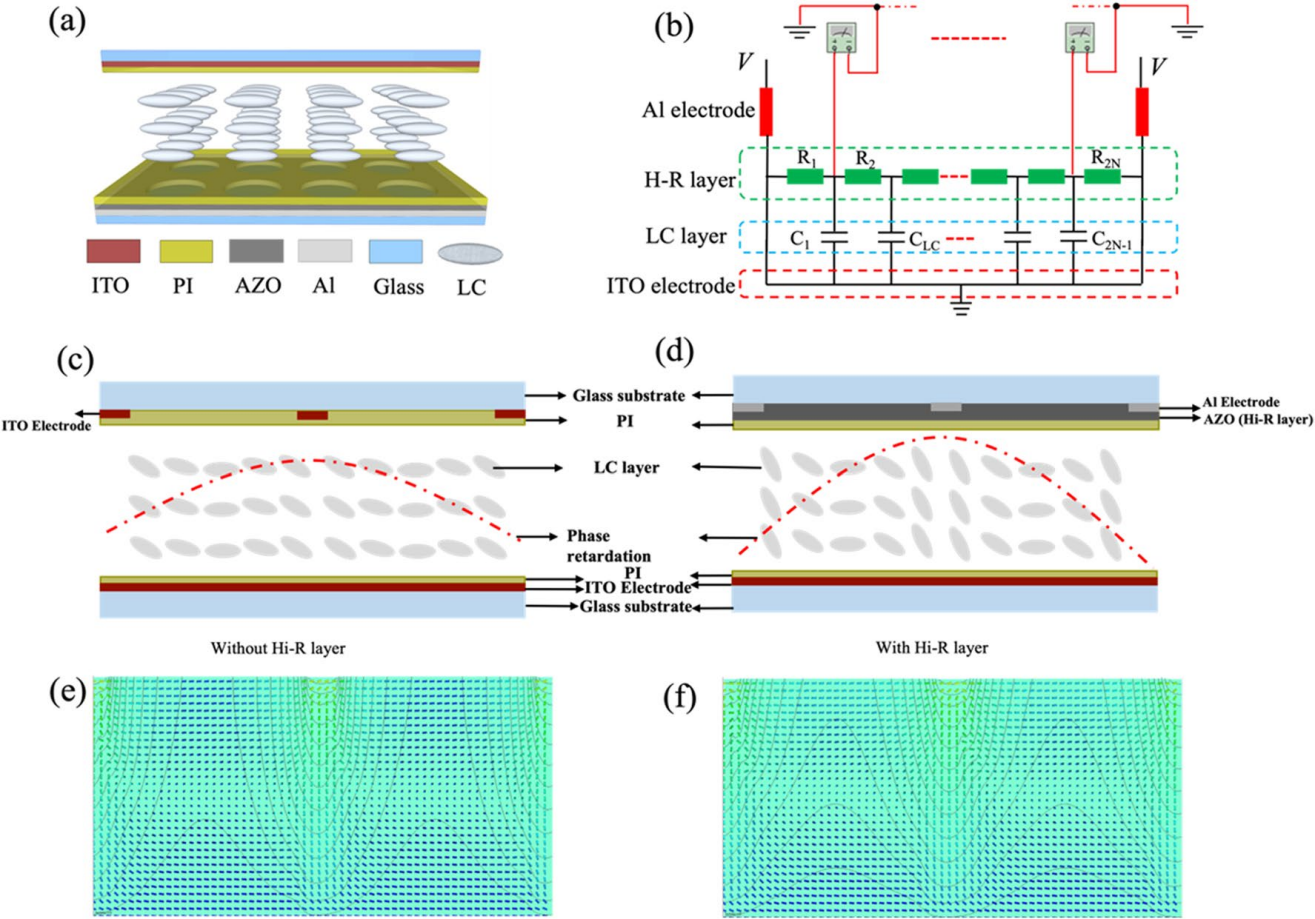
(**a**) Section view of the proposed structure; (**b**) R–C circuit model of the proposed structure; (**c**) schematic diagram of phase retardation of LC MLAs without the Hi-R layer; (**d**) schematic diagram of phase retardation of Hi-R LC MLAs; (**e**) equipotential line and direction distribution of LC MLAs without the Hi-R layer and (**f**) equipotential line and direction distribution of Hi-R LC MLAs.

Figure 7a shows a section vies of Hi-R LC MLAs, which mainly consists of an LC layer, hole-patterned electrodes with Hi-R layer, PI layer, an ITO flat electrode and substrates. When an working voltage is applied between the hole-patterned electrodes and the ITO flat electrode, an electric field can be generated gradually from the edge to center of the patterned hole and formed the LC MLAs. However, the working voltage supplied to the LC MLAs must be raised until a gradient electric filed is formed inside each LC lens. Therefore, a transparent aluminum-doped zinc oxide (AZO) film is deposited on the the hole-patterned electrodes as Hi-R layer to decrease the working voltage of LC MLAs.

An R–C circuit model was established by using Multisim software to determine the optical characteristics of the LC MLAs and the effection of Hi-R layer on their performance. This model is mainly composed of the AZO layer in a LC structure, as shown in Fig. 7b. The Hi-R layer is separate into *N* (N ≥ 10) parts, and the resistance of each part is equal in a single LC lens, which is deposited on the surface of each hole-patterned electrode. Accordingly, it will induce a gradient voltage distribution on the nodes of the R–C circuit as an alternating current (AC) voltage is applied to the LC MLAs with Hi-R layer. Therefore, the potential difference in the LC cell is easily generated a parabolic phase retardation under the role of the Hi-R layer compared with the traditional LC structure, as depicted in Fig. 7c, d, respectively.

To investigate the director distribution and the electric field distributions of LC molecules in the LC cell in the working state, TechWiz LCD 3D software was employed to simulate the LC MLAs without and with Hi-R layer. For the LC MLAs without the Hi-R layer, the equipotential lines are mainly concentrated on the edge of each hole-patterned electrode, as shown in Fig. 7e. For the given voltage, the LC molecules in the center of the lens are arranged along the rubbing direction because of the weaker electric field, which is difficult to form the LC lens. For the LC MLAs with Hi-R layer, the Hi-R layer is very essential to generate linearly varying electric potential from the LC lens center to the edge and smoothen the phase profile of the LC lens, as plotted in Fig. 7f. Meanwhile, owing to the existence of the Hi-R layer, the proposed LC lens array will not induce any disclination lines in the uniform LC layer to cause light scattering which degrades the optical performance of the lens because the generated electric fields are in the same vertical directions compared with an LC lens with an inside hole-patterned electrode, and the equipotential lines generated at the same voltage spreads from the edge to the center of the hole-patterned electrode easily under the role of the inserted AZO Hi-R layer^42^. The deflection angle of the LC director increases gradually from the center to the edge until nearly perpendicular to the substrate, which indicates that a refractive index with a gradient distribution can be generated. These simulation results demonstrate that the Hi-R LC MLAs is easier to acquire the uniform gradient drop from the center to the edge at the same voltage by contrast to the LC MLAs without the Hi-R layer. A stronger electric field usually results in a larger deflection angle *θ* of the LC directors. The effective refractive index can be given by using the following equation:

3 $$n_{eff} (\theta ) = n_{o} n_{e} /(n_{o}^{2} \sin^{2} \theta + n_{e}^{2} \cos^{2} \theta )^{1/2}$$

where *n*_*o*_ and *n*_*e*_ denote the refracitve didex for the ordinary light and extraordinary light, respectively. And *θ* denotes the deflect angle between the LC optical axis and the polarization of incident light.

### Fabrication of Hi-R LC MLAs

As illustrated in Fig. 7a, Hi-R LC MLAs are consist of a ITO common electrode within the upper substrates, LC layer and a bottom glass substate with a hole-patterned electrode arrays and a Hi-R layer. The fabrication process of Hi-R MLAs mainly includes two steps: the fabrication of Al hole-patterned electrode arrays with an aluminum-doped zinc oxide (AZO) Hi-R layer and the assemble of LC lens. Firstly, an ~ 100 nm aluminium (Al) layer was deposited on the bottom glass substrate by magnetron sputtering. The hole-patterned electrode arrays were prepared on the bottom glass substrate by a photolithography and wet-etching process. After that, an ~ 30 nm AZO film was deposited on the surface of the hole-patterned electrode arrays at an Ar flow rate of 10 SCCM, sputtering pressure of 5.8 × 10^–2^ Pa and radio frequency power of 1000 W.

Secondly, the Hi-R LC MLAs were successfully fabricated by employing the delicate designed LC cell structure. After curing at 260 °C for 40 min, PI solution were coated on the surface of Hi-R layer and common electrode by a spin-coating method, which is considered as an orientation direction of LC molecules was obtained. It was notable that the rubbing directions of PI layers were anti-parallel so that LC molecules in the LC cell can uniformly distribute. The glues were printed along the edge of the top substrate by a screen printing and the LC cell was separated by the spacers with a diameter of 45 μm. And then, the upper and bottom glass substrates were placed anti-parallel to ensure a uniform distribution of LC molecules along the rubbing direction. The Hi-R LC MLAs were successfully fabricated after the LC materials (Merck E7, *n*_*o*_ = 1.525, *n*_*e*_ = 1.748, and *Δn* = 0.223) were injected into the LC cell and the seaming hole was sealed with the glue.

## Data Availability

All data generated or analysed during this study are included in this published article.
